# Partisan Bias in Message Selection: Media Gatekeeping of Party Press Releases

**DOI:** 10.1080/10584609.2016.1265619

**Published:** 2017-07-03

**Authors:** Martin Haselmayer, Markus Wagner, Thomas M. Meyer

**Keywords:** election campaign, political parties, media gatekeeping, partisan bias, news value

## Abstract

Parties try to shape media coverage in ways that are favorable to them, but what determines whether media outlets pick up and report on party messages? Based on content analyses of 1,496 party press releases and 6,512 media reports from the 2013 Austrian parliamentary election campaign, we show that media coverage of individual party messages is influenced not just by news factors, but also by partisan bias. The media are therefore more likely to report on messages from parties their readers favor. Importantly, this effect is greater rather than weaker when these messages have high news value. These findings have important implications for understanding the media’s role in elections and representative democracies in general.

## Introduction

Political actors produce a large number of messages, but not many of these are likely ever to reach the public. In part, this is because citizens are still mostly exposed to political actors’ messages via the news media, but these can only present consumers with a sample of all messages from political actors. Hence, much of what citizens are able to read, see, and hear about political actors is ultimately determined by journalists and editors. This is known as media gatekeeping (Shoemaker & Reese, 1996)—that is, “the process by which countless occurrences and ideas are reduced to the few messages we are offered in our news media” (Shoemaker & Vos, 2009, p. 75). In this article, we study which individual messages from political actors are reported on by the media during an election campaign, focusing on two aspects—partisan bias and news value—and how they interact.

We argue that media outlets are systematically more likely to sample messages from some political actors than from others because they have a partisan bias toward specific parties (Puglisi & Snyder, 2011). Such partisan gatekeeping bias may be driven by supply-side or demand-side factors. On the supply side, political parallelism is likely to be a key contributing factor (Hallin & Mancini, 2004): if some media outlets are close to specific parties in ideological, organizational, and institutional terms, those outlets may report more about those actors and be more responsive to their messages. On the demand side, media outlets will differ in the political actors their readers support, and it may be that they try to provide more and better coverage of the actors favored among readers (e.g., Gentzkow & Shapiro, 2006; Mullainathan & Shleifer, 2005).

The second aspect we study—news value—is a well-established predictor of media coverage. News value refers to the overall newsworthiness of a message and can be defined by the presence or absence of a number of news factors (Staab, 1990). News factors that are particularly important for the coverage of politics and election campaigns include the role of power elites, conflict, surprise, the relevance of the topic, and its continuity in the media issue agenda (see Helfer & Van Aelst, 2016; Hopmann, Elmelund-Præstekær, Albæk, Vliegenthart, & Vreese, 2012; Tresch, 2009). In other words, journalists may be more likely to report on messages from important politicians that include new information on important topics the media is already reporting on and that other political actors are discussing.

We add to this research on news value by arguing that partisan bias and news value interact. The reason for this conditionality is the imbalance between the sheer amount of political messages and a very limited media agenda. As the number of messages from political actors usually exceeds by far the space newspapers allocate to political coverage, partisan bias alone is an insufficient selection criterion. The consequence of this is that the effect of partisan bias is even more visible for messages with high news value. The interaction between news value and partisan bias also has implications for our understanding of how bias in political news coverage impacts on voter opinions and their vote choices (Druckman & Parkin, 2005; Eberl, Boomgaarden, & Wagner, 2015; Hopmann, Vliegenthart, De Vreese, & Albæk, 2010), particularly as we show how and why the information voters receive may differ systematically between media outlets.

Our empirical analysis of how partisan bias determines the media coverage of individual messages is based on original party and media data on the 2013 Austrian general election. Following Hallin and Mancini (2004), the Austrian media system is similar to those in other northern and central European countries (Belgium, Germany, the Netherlands, Scandinavian countries, and Switzerland). This means that, on the one hand, Austria is a likely case to find partisan bias in newspaper reporting as there is a relatively high level of polarization in party and media systems. On the other hand, it is usually assumed that the partisanship of these European media systems has faded, so finding evidence of partisan bias is substantively relevant for our understanding of the contemporary relationship between media and politics.

We measure whether party messages in press releases make it into media reports throughout eight newspapers in the final six weeks of the 2013 Austrian election campaign. Like Grimmer (2010, 2013), we use cheating detection software to compare 1,496 relevant press releases with 6,512 media reports. Once the software has narrowed the number of potential text pairs, we manually code whether or not a media report is based on a particular press release. We supplement this information with survey data that link media outlets to their readers’ party preferences.

Our analysis adds to a growing literature that analyzes media gatekeeping for individual party messages. Previous research has considered party issue agenda-setting in the media (Brandenburg, 2002, 2006; Hopmann et al., 2012) or the visibility of political actors in the news (Cook, 1988; Gattermann & Vasilopoulou, 2015; Kriesi, 2012; Tresch, 2009). More recent studies have focused on media gatekeeping of individual messages such as parliamentary questions (Van Aelst & Vliegenthart, 2014; Van Santen et al., 2015) and party pledges (Kostadinova, 2015). So far, little research on gatekeeping has looked at press releases, with a few exceptions from the United States (Flowers, Haynes, & Crespin, 2003; Grimmer, 2010, 2013) and Europe (Helfer & Van Aelst, 2016).

Our findings concerning partisan bias have important implications for understanding the role of the media in democracies. Media outlets are biased in the types of messages they report on, privileging those political actors their readers prefer. This gatekeeping bias is most prevalent for party messages with a high news value. This also implies that news value alone is not always sufficient for party messages to “make the news.” In the conclusion, we consider in more detail the relevance of these findings for party and media systems in other countries.

## Determinants of Media Gatekeeping

**Figure 1.**
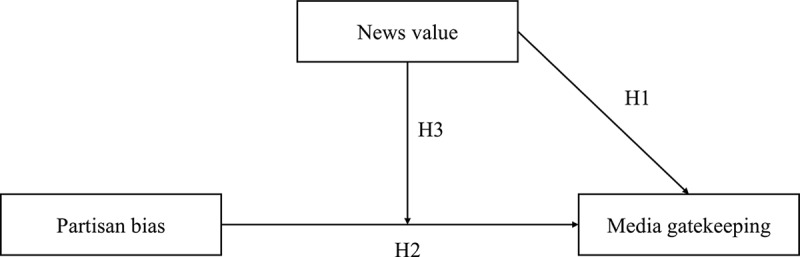
Determinants of media gatekeeping.

We propose a model of when journalists report on messages from political actors as sources in their news reports (Figure 1). We aim to explain the success of individual messages (such as press releases) in being reported on in the media. This approach differs from analyses that examine which issues political actors are associated with in the media (e.g., Hopmann et al., 2012). These “mediated issue appeals” may be influenced by many actors (such as political rivals), while we focus on the success of individual messages in getting media coverage.

The model builds on two explanatory variables: news value and partisan bias. Thus, journalists are more likely to report on political messages if they have a higher news value (Hypothesis 1). Moreover, journalists are more likely to pay attention to messages from political actors they (and their readership) favor (Hypothesis 2). Finally, we hypothesize that these two factors interact in determining media selection of political messages: the vertical arrow in Figure 1 indicates that the effect of partisan bias on media reporting is conditional on the news value of the political message. Thus, journalists are particularly likely to pick messages of political actors they favor if those messages also have a high news value (Hypothesis 3).

### The Effect of News Value

Stories and events differ in their underlying newsworthiness, and this should also explain the extent to which they are reported on (Galtung & Ruge, 1965; Harcup & O’Neill, 2001; Helfer & Van Aelst, 2016; O’Neill & Harcup, 2009). The underlying idea is that journalists and editors have a common understanding of factors that make a message newsworthy (Helfer & Van Aelst, 2016). The more these factors are present in a message, the higher its newsworthiness, and thus the greater its chances of being covered (Staab, 1990). However, what exactly these news factors are is a matter of debate and may depend on the kinds of news that are analyzed and what other news competes for the journalists’ attention (Staab, 1990). We follow previous research in identifying five news factors that are particularly important for coverage of political actors (see Helfer & Van Aelst, 2016; Hopmann et al., 2012; Tresch, 2009): political power, negativity, surprise, relevance, and continuity.

First, we expect messages by powerful politicians to have higher chances to make the news than those sent by actors without a powerful position (Balmas, Rahat, Sheafer, & Shenhav, 2014; Entman, 2007; Galtung & Ruge, 1965; Gans, 1979; Helfer & Van Aelst, 2016; Tresch, 2009; Van Aelst, Maddens, Noppe, & Fiers, 2008; Vliegenthart, Boomgaarden, & Boumans, 2011). Second, the media is particularly attentive to stories that include negativity and conflict (e.g., Ansolabehere & Iyengar, 1995), so campaign messages that satisfy the media’s demand for conflict and aggression should carry a higher news value (Gans, 1979). Third, unexpected or surprising messages should make the news more easily than routine stories. This news factor has been termed unexpectedness or surprise (Galtung & Ruge, 1965; Harcup & O’Neill, 2001). Journalists may thus look for messages where parties discuss issues they usually emphasize less (Helfer & Van Aelst, 2016). Fourth, the media should be more likely to cover stories that are relevant; in political news, such relevance may be indicated by whether an issue is important to several political actors, which also helps the media in striving for balance (Green-Pedersen, Mortensen, & Thesen, 2017; Van Aelst & De Swert, 2009; Walgrave & Van Aelst, 2006). Thus, the more parties address an issue, the more likely a campaign statement should make the news. Finally, continuity should enhance the chances of a political message to make the news, as the media have an interest in adding to an existing story (Allern, 2002; Galtung & Ruge, 1965).

All of the news factors just presented should increase a message’s news value, and messages with more news factors should be more likely to be used as a source in media reporting. Thus, we hypothesize:

H1 (News value): Press messages with higher news value are more likely to be used as a source in media reports.

### The Effect of Partisan Bias in the Media

In addition to news value, partisan bias may also affect whether the media report on messages of political actors. More specifically, media coverage of campaign messages may be more favorable to some political actors than to others. For example, partisan media tend to deemphasize negative stories or scandals about political actors they favor and overemphasize the faults of disliked ones (Baumgartner & Chaqués Bonafont, 2015, p. 269; Puglisi & Snyder, 2011).

The reasons why they favor one political actor over another may vary. Puglisi and Snyder (2011) distinguish supply-side factors, such as the political preferences of editors and journalists, from demand-driven factors, such as reader preferences. Supply-side factors may reflect an institutionalized bias within the media system, a concept captured by the term *political parallelism*. Political parallelism describes how the political system and the media system are tied to each other and how well different political views are represented in the news (Hallin & Mancini, 2004, p. 26ff.). The greater political parallelism is, the more likely journalists may be to exhibit bias in their reporting. This bias may be an overt partisan slant, but in a weaker form, it may lead to a gatekeeping bias and selective emphasis in news reporting (Hallin & Mancini, 2004). Demand-driven factors may shape reporting if newspapers know that their readers on average support some political actors more than others and prefer to read news about them (e.g., Iyengar & Hahn, 2009). For instance, party supporters may want to know how their party reacted to a legislative proposal. Responsive media may decide to selectively cover messages from political actors that are popular among their readership. This is largely a demand-based account of media gatekeeping since it originates among readers rather than among media actors (e.g., Gentzkow & Shapiro, 2006; Mullainathan & Shleifer, 2005).

In the empirical analysis that follows, we use a demand-side-driven measure to study partisan bias. Ideally, we would also measure supply-side-driven support, which is usually captured using a newspaper’s endorsement of a party or candidate (e.g., Puglisi & Snyder, 2011). While such endorsements are quite common in Anglo-Saxon countries, support for a specific party is less open in Austria. This makes it hard to find appropriate supply-side measures of partisan bias. Moreover, Puglisi and Snyder (2011) report a modest positive correlation between both measures in the United States, and weaker effects for the demand-side-driven measure. Thus, our operationalization might be considered as a conservative test of our hypothesis.

Our second hypothesis is therefore the following:

H2 (Partisan bias): Media outlets are more likely to report on press messages from political actors their readership favors.

### News Value As a Moderator of the Partisan Bias Effect

Finally, we are interested in the moderating effect of news value on the impact of partisan bias. It could be that friendly media choose to focus on messages from favored political actors even if these messages contain little in the way of news value. However, election campaigns are very busy periods where political actors are in a constant contest for media attention. In Austria, politicians from the major parties issued hundreds of press releases in the last six weeks of the election campaign. Even ignoring other campaign events such as TV debates or campaign rallies, the sheer amount of messages by political actors that aim for media attention is often in stark contrast to the limited media agenda. Therefore, a journalistic heuristic to focus on messages by favored political actors would to some extent be insufficient, as even the most favored actors send more messages than a media outlet could report on. Thus, editors and journalists need to ignore most messages, even those from political actors they prefer the most (e.g., Meyer, Haselmayer, & Wagner, 2015; Van Aelst & Vliegenthart, 2014; Van Santen et al., 2015).^1^ When choosing among messages from the same political actor, journalists and editors will therefore prefer to report on campaign messages that have the additional benefit of containing (higher) news value. Thus, we expect a positive interaction between readership party orientation and news value: media outlets should be most likely to report on press messages that have a high news value *and* are from a political actor they favor.

We therefore test whether partisan gatekeeping bias is most likely for messages with high news value.^2^ The consequence of this would be that the readers’ party orientation matters more when there are several news factors, meaning that the biasing effects of gatekeeping are greater precisely when messages have a higher news value. Our final hypothesis is therefore the following:

H3 (Partisan bias | News value): Media outlets are more likely to report on messages from political actors their readership favors, and this effect increases as a message’s news value increases.

## Data and Methods

We study media gatekeeping by comparing newspaper articles with party campaign messages as contained in their press releases. The empirical analysis is based on content analyses of party press releases and newspaper articles published in the last six weeks of the 2013 Austrian national election campaign. Focusing on one country (and a single campaign) allows us to study the success of campaign messages based on the full universe of party messages and media coverage. Such a strategy is crucial for our research question, because restricting the coverage of media outlets would affect our estimates of the success of press releases.

Austria is a parliamentary, multiparty system that shares many characteristics with those in other European countries. It is characterized by moderate pluralism (Sartori, 1976) with a center-left (SPÖ) and a center-right (ÖVP) party. The Greens on the left and the Freedom Party (FPÖ) on the right were the main opposition parties in parliament. The BZÖ, a splinter party founded in 2005, lost most of its popularity between the general election in 2008 and the 2013 election campaign. Some MPs left that party and joined the movement of billionaire Frank Stronach (Team Stronach).^3^

According to Hallin and Mancini (2004), the Austrian media system shares similarities with a number of northern and central European countries (Belgium, Germany, the Netherlands, Scandinavian countries, and Switzerland). There are quality newspapers and tabloids, and some of them also have a strong regional focus. Austria is also a country where print editions of newspapers are still highly relevant: about 73% of the population (above 14 years of age) read newspapers on a daily basis (Aichholzer et al., 2014, p. 32), which makes them attractive targets of party campaign communication and increases the potential effects of successful party communication. Focusing on paper editions of newspapers further allows us to establish a closer causal link between campaign messages at day *t* and media content at day *t + 1* (see later discussion). The news cycle of TV broadcasts or online news would make it much harder to establish similar links for these media. Finally, data from the Austrian National Election Study (AUTNES) allow us to match these party data with the media coverage of all major national newspapers and of the public issue agenda.

Press releases are a particularly useful means of capturing what parties want the media to talk about (e.g., Brandenburg, 2002; Grimmer, 2010; Hopmann et al., 2012). We focus on press releases distributed by parties represented in parliament (SPÖ, ÖVP, FPÖ, Greens, BZÖ, and Team Stronach). These parties published 1,922 party press releases in the final six weeks of the campaign. We drop press releases informing journalists about party campaign events (e.g., press conferences, photo ops) and those merely containing pictures and hyperlinks to audio content (*N* = 105). To study the effect of power elites, we identify the politician who issued the press release. Some press releases are sent by two politicians, and these press releases enter the analysis separately for each politician.^4^ We also exclude those releases where only a party label (and not an individual politician) is provided as the sponsor of the press release because they cannot be used to test hypotheses about sender attributes (*N* = 195). Moreover, we discard releases that are not policy-related and, for example, merely contain information on specific campaign events (TV debates, canvassing), opinion polls, and changes in party office (*N* = 247). The remaining press releases are assigned to 18 issue areas by aggregating codes from a coding scheme with more than 650 categories.^5^ This aggregation is necessary to avoid excessive zeros for many of these issues and to merge these data with mass survey data on voter perceptions of party competence. In total, this leaves us with 1,496 campaign messages published by 292 individual politicians.

### Dependent Variable

We measure media gatekeeping by analyzing whether there is at least one media report in a newspaper using a press release as a source (1) or not (0). This operationalization follows that in previous research on the media coverage of press releases in the United States (Flowers et al., 2003). We group press releases by day, creating 41 clusters of press releases. Next, we identify media reports in eight daily newspapers (*Der Standard, Die Presse, Salzburger Nachrichten, Kronen Zeitung, Österreich, Heute, Kurier*, and *Kleine Zeitung*) the day after the press release was sent.^6^ To avoid bias, our sample captures media reports from all newspaper sections. Specifically, we use headlines, media reports, and background analyses but exclude other types of media reports such as commentaries, interviews, cartoons, and letters to the editor (*N* = 6,512).

Each press release needs to be checked against an average of roughly 170 media reports published the following day, meaning that there are about 270,000 press release-media report dyads. To handle these data, we follow Grimmer (2010) and employ a two-stage coding process. First, cheating detection software (Bloomfield, 2014) is used to identify media reports with content that overlaps with that of a press release published the day before. The goal of this automated analysis is to narrow the number of coding units for the following hand-coding process. Yet, to avoid false negatives (e.g., successful press releases not detected by the software), we choose non-restrictive settings to generate more “hits.”^7^ The cheating detection software identifies 1,785 potential pairs of text, thus allowing us to discard 99.5% of all press release-media report dyads.

Next, we go through these text pairs manually, reading the press release and the media report, to assess whether a press release was successful in making the news. A coder deemed a press release as successful if the media report (a) named the press release’s author (i.e., name or party label) as an active speaker in the article and (b) dealt with the same topic as the press release. Examples of “successful” press releases are shown in supplemental Appendix B.

Since manually coding the success of press releases is not always straightforward, we assess the reliability of the manual coding process by having a sample of 500 coding decisions made by two coders. The intercoder reliability using Krippendorff’s alpha is 0.82 and thus reasonably high. We also checked coder decisions and found disagreement often occurred when press releases and media reports refer to a third event (e.g., a press conference). Actors holding public or party office are more likely to give press conferences, and we might therefore overestimate their success in getting media coverage for their messages. This is why we add a control variable to our models that indicates whether a press release refers to statements made at a press conference.

### News Value

To identify the news value of each press release (H1), we build a news factors index ranging from 0 to 5 that builds on five news factor dummy variables. To measure *powerful actors*, we use a binary indicator of whether a press release was issued by one of the following: a member of government, a party leader, or a party chairperson.^8^ We measure *conflict* based on whether a press release includes a negative statement about a rival party or politician. To measure *surprise*, we code the parties’ issue competencies using a rolling cross-section voter survey carried out during the campaign (Kritzinger, Johann, Aichholzer, et al., 2014). The share of respondents naming a particular party as being best to handle an issue is used as a measure for the party’s competence. For each party, we use a median split of their competence scores to distinguish their “best” issues from the rest. The dummy variable “surprise” is “1” if a party addresses an issue in a press release that is not among its best issues, and “0” otherwise. To measure the *relevance* of an issue in a press release, we code the number of parties addressing an issue on a specific day. The measure ranges from 1 (one party addresses an issue) to 6 (all parties address an issue). We use a median split to distinguish more relevant from less relevant press releases. Finally, we measure the *continuity* of a news story by calculating the daily media issue agenda as the share of newspaper articles in a media outlet dealing with the respective issue area on the day a press release was published. As mentioned earlier, we use a median split for the index to distinguish issues high on the media’s issue agenda from less salient ones.

The news value index is an additive index of the five news factors. This assumes that the five news factors have a similar impact on a message’s news value. To test the robustness of our results we reran the analysis using different indicators of a message’s news value. The results (reported in supplemental Appendix D) are similar to the ones reported here.

### Readership Party Orientation

As a measure for partisan bias (H2), we rely on political preferences of a news medium’s readership measured in an online survey (Kritzinger, Johann, Glantschnigg, et al., 2014). Participants in an online access panel by TNS Opinion were randomly selected to take part in the survey based on key demographics (age, gender, region, and household size); the final sample closely resembles the Austrian population on these characteristics. About 3,000 respondents were asked to indicate which newspapers they read. Among all readers of a media outlet, we measure *partisan preferences* for each party using a respondent’s self-assessed probability of ever voting for a party (from 0 to 10).^9^

**Figure 2.**
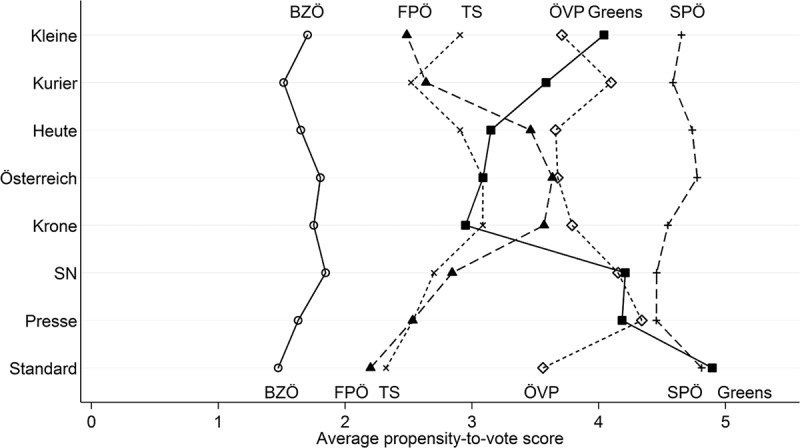
Readership party orientation across media outlets. *Notes*: Average propensity to vote for six parties (SPÖ, ÖVP, FPÖ, Greens, TS, and BZÖ) by readership of eight Austrian newspapers. Higher values indicate a higher propensity to vote for the respective party, and thus a more favorable partisan bias. Data: Kritzinger, Johann, Glantschnigg, et al. (2014).

Figure 2 shows the readership party orientation (x-axis) for eight Austrian media outlets (y-axis). It is important to keep in mind that the measure of readership party orientation indicates readers’ average propensity to vote for a party. Thus, a score of “5” indicates that the average reader states that there is a 50% chance they will ever vote for that party, which is arguably a rather high probability in a multiparty system. Keeping this in mind, there is considerable variation in the media’s readership orientation toward specific parties. The Freedom Party (FPÖ), for example, has a much better standing among readers of tabloid newspapers (*Heute, Kronen Zeitung, Österreich*) than in other media outlets. In contrast, the readership party orientation toward the Greens is much more positive in quality newspapers like *Der Standard* or *Die Presse* than in tabloids like *Österreich*. There are also party differences that are rather stable across media outlets. For example, readership party orientation is generally more favorable toward the SPÖ than for the BZÖ. Because these overall party-level differences may capture differences in the parties’ newsworthiness, we include party fixed effects in the multivariate analysis.

### Control Variables

There are several control variables that need to be taken into account. First, we control for whether press releases sent by women (1) are less successful than those sent by men (0). Furthermore, we include a variable indicating the *time* a press release was published. Press releases published in the late afternoon or in the evening may have fewer chances to get the media’s attention than those published in the morning. The variable indicates the time (in minutes) since midnight. Similarly, we control for the *date* a release was sent out: there are more press releases later in the election campaign, so getting into the news is more difficult then. It is a count of the day in the election campaign, and thus increases as the election draws near. We also include a variable specifying whether a press release is based on an *external event* (1) or not (0). This variable is based on a broader measure (included in the AUTNES data set) that captures the trigger of a press release. We consider a press release as being triggered by an external event if it is based on an external event in the international arena (e.g., a European Union summit) or stemming from national actors outside the party and media arena (e.g., a report of the Austrian audit court). We also measure whether a press release refers to *press conference* (1) or not (0). Moreover, we account for the (total) *number of articles* in a media outlet over the six-week period because more articles provide more opportunity for a press release to be successful in a specific media outlet. We also control for the *text length* (in words) of the press releases to account for the level of information they contain. Finally, we also include party fixed effects to capture any remaining variation among parties.

### Model Specification

We use logistic regression models since our dependent variable indicates whether a press release is reported on (1) or not (0). We test whether press releases (*N* = 1,496) are reported in eight media outlets, meaning that the data are “stacked,” as there are eight observations for each press release (*N *= 11,968). Yet, these observations in our data set are not independent as press releases are clustered in parties and each press release may be reported on in eight different media outlets. To account for this cross-classified data structure, we calculated intra-cluster correlation coefficients using different model specifications for clusters (e.g., party level, media outlets, party-media pairs, press releases). Most of the variation is located in party press release clusters. Thus, we use logistic regression models with standard errors clustered by party press releases. To check the robustness of our results, we also report results for multilevel logistic regression models with random intercepts (at the level of press releases) in supplemental Appendix C. The effects are similar to (albeit smaller than) the ones reported here.

## Results

**Figure 3.**
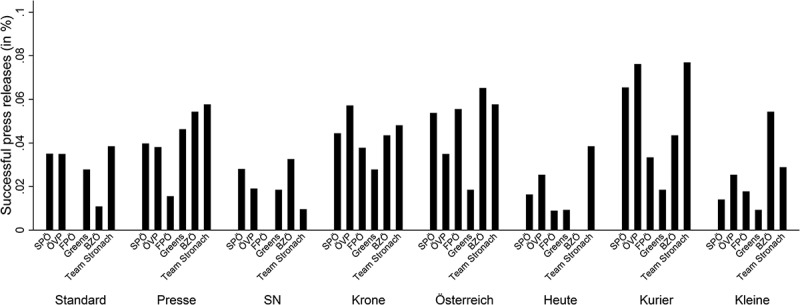
Share of press releases in media reports, by media outlets and parties. *Note*: Bars denote the average success rate of press releases across media outlets and parties.

Figure 3 shows the share of successful party press releases across parties and media outlets. There is substantial variation in the success of party press releases in making the news. For example, press releases by the radical right Freedom Party (FPÖ) are more likely to be covered in tabloid outlets (*Österreich* and *Kronen Zeitung*) than in broadsheets such as *Der Standard* or *Die Presse*. In contrast, the Greens’ press releases are most likely to be covered in *Der Standard* and *Die Presse*, while their success rate is somewhat lower for tabloid and midrange papers. There is also important variation across media outlets, as some of them report more on party press releases than others do: the success rates range from 1.6% of all press releases (*Heute* and *Salzburger Nachrichten* [*SN*]) to 5.4% (*Kurier*). This might be due to the space media outlets allocate to coverage of political news. We account for these differences in the multivariate analyses by including the number of articles per media outlet as a control variable.

Table 1 presents the regression results of two logistic regression models. Model 1 shows the unconditional effects of news value (H1) and readership party orientation (H2) on media gatekeeping. Model 2 includes an interaction term to test whether the effect of readership party orientation is conditional on a message’s news value (H3).

**Table 1:** Explaining the success of press releases

	Model 1	Model 2
News value index	0.325***	–0.364^+^
	(0.068)	(0.211)
Readership party orientation	0.515**	0.0435
	(0.157)	(0.222)
Readership party orientation X News value index		0.188**
		(0.060)
Female	–0.240	–0.214
	(0.193)	(0.194)
Time press release (PR) sent	–0.00116^+^	–0.00123*
	(0.001)	(0.001)
Date	–0.0181***	–0.0184***
	(0.005)	(0.005)
PR based on external event	0.0617	0.0681
	(0.185)	(0.185)
PR with press conference	1.204***	1.169***
	(0.207)	(0.210)
Number of articles per newspaper	0.000811***	0.000818***
	(0.0001)	(0.0001)
Text length	0.00252***	0.00253***
	(0.001)	(0.001)
Constant	347.6**	355.5***
	(106.661)	(105.107)
Observations	11968	11968
Log likelihood	–1553.4	–1545.9

*Notes*. Clustered standard errors (for press releases) in parentheses (1,496 clusters).

Party fixed effects included but not reported in the table.

^+^ *p* < 0.1. * *p* < 0.05. ** *p* < 0.01. *** *p* < 0.001.

Model 1 indicates that a message’s news value has a positive effect on the probability that a press release is successful (H1). Model 1 also shows support for the effect of readership party orientation on the coverage of party press releases in media reports: the more favorable a media outlet’s readership is toward a party, the more likely it is that a press release from that party is used as a source in a media report (H2).

**Figure 4.**
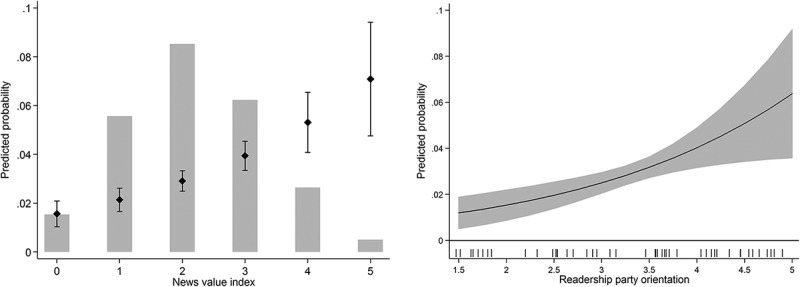
The effects of news value and partisan bias on the success of press releases. *Notes*: Y-axes show the predicted probability that a party press release gets media attention along with 95% confidence intervals. The x-axes show the distribution of news value (H1; left panel) and readership party orientation (H2; right panel). The bar chart (left panel) and the pipes (right panel) indicate the distribution of both variables. Estimates based on Model 1 in Table 1. All remaining variables are held constant at their observed values.

Figure 4 illustrates both effects in terms of predicted probabilities (y-axis). Bars show the distribution of news values in party messages in the left panel, while the pipes in the right panel illustrate the distribution of party media orientation. Predicted probabilities are reported along with 95% confidence intervals. Both news values and readership party orientation significantly increase the chances that a press release is successful in getting reported. Moreover, the magnitude of both effects is quite similar: increasing the news value from 0 (minimum) to 5 (maximum) increases the probability to get into the media from 1.5% to 7%. Similarly, changing the readership party orientation from the most hostile (1.5; e.g., BZÖ in *Kurier*) to the most favorable (5; e.g., Greens in *Der Standard*) readership party orientation increases the probability of being reported on in the media from 1.2% to 6.4%.

We use two tests to check the robustness of these results. First, we check whether the results for H1 are driven by a particular news factor by rerunning our analysis excluding one news factor at a time (results shown in supplemental Appendix D). The substantial conclusions do not change: news factors have positive impact on the success of party press releases in all model specifications. Second, we also test whether the effect of readership party orientation hinges on differences across media outlets or political parties. Some parties are particularly popular (SPÖ, ÖVP) or unpopular (BZÖ) among readers of all media outlets (see Figure 2). We therefore reran the analysis excluding these parties. The results based on this model are very similar to the ones presented here (results shown in supplemental Appendix E).

Turning to the conditional effects, Model 2 in Table 1 shows the results for the moderating effect of news value on the impact of readership party orientation. We find that press releases are reported on more if they face a favorable news outlet *and* have a higher news value (H3). Note that the two constituent terms are the effects of each variable if the other is 0, so the statistically significant negative effect of news values should not be interpreted directly.

**Figure 5.**
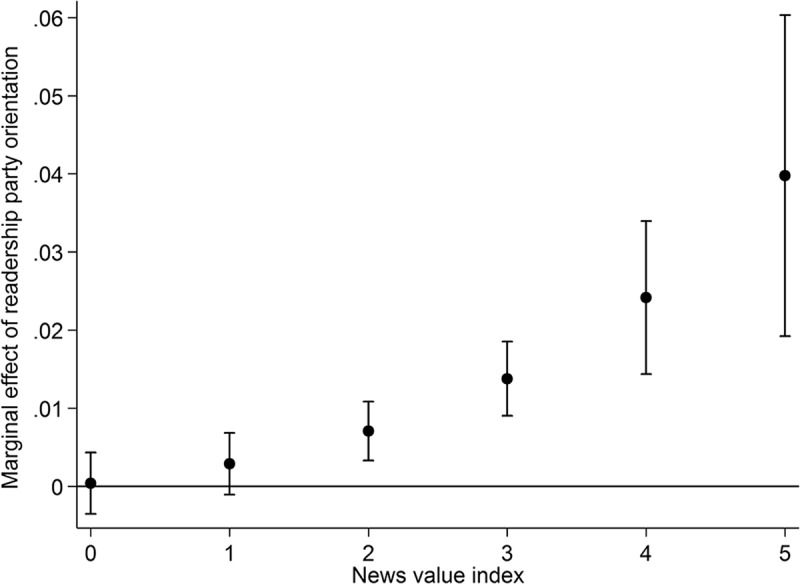
Marginal effects of partisan bias conditional on news value. *Notes*: Marginal effects are based on Model 2 (Table 1) holding all other variables at their mean or mode. Whiskers indicate 95% confidence intervals.

Figure 5 shows the marginal effect of readership party orientation for different levels of news factors. Thus, unlike the predicted probabilities in Figure 4, positive estimates indicate that readership party orientation has a positive effect on the success of party press releases. The results in Figure 5 show support for the moderating effect of news values for the effect of media partisanship: Press releases are more likely to be covered in media outlets that are favorable to them, but this effect is greater if the messages have a higher news value. In fact, the marginal effect for the least newsworthy press releases is close to zero and not statistically significant at conventional levels. This means that media partisanship plays little or no role for the least newsworthy press releases. Instead, partisan bias matters most for press releases by powerful politicians and those dealing with issues that are surprising, contain conflict, are relevant for other parties, or already present in the media.^10^

## Conclusion

We find that partisan bias affects the micro-level coverage of individual party press releases by the media. Our study is among the first observational studies to demonstrate the influence of such partisan bias in a general election campaign. Moreover, we find that partisan bias is strengthened by the presence of news factors: when press releases have a higher news value, the impact of partisan orientation on media coverage increases. This is important for understanding the relevance of gatekeeping bias. It is not the case that partisan bias means that some media outlets report on certain political actors’ messages regardless of their news value. It is also not the case that high news value cancels out the effects of partisan bias. Instead, the likelihood that messages with a high news value are reported on is greater if the message is from an actor the outlet is biased toward. Hence, it is particularly on newsworthy messages that partisan bias in message selection takes on importance. This is especially relevant as this pattern increases the substantive importance of bias in message selection, as media outlets will diverge particularly in their coverage of substantively newsworthy political messages.

The existence of partisan gatekeeping bias is important: for instance, unbalanced coverage of politics and media choice may reinforce differences in the distribution of political knowledge and increase the political polarization of opinions among citizens (e.g., Levendusky, 2013; Prior, 2005, 2007). Ultimately, biased coverage may also affect vote choice, even if these biases are only present during short-term election campaigns (Druckman & Parkin, 2005; Eberl et al., 2015). An effect on vote choice is plausible because readership party orientation varies but is not perfectly aligned with each outlet. In other words, there are many potential “converts” even among readers of newspapers with a particularly strong partisan following.

In terms of media systems, the fact that we find evidence of partisan bias is significant particularly because research on political parallelism has predicted a steady decline of partisanship as media systems converge toward a liberal (and commercial) model characterized by high professionalism based on principles of “objectivity” and political neutrality (Hallin & Mancini, 2004). However, if partisan bias remains, then this may indicate that heightened economic competition exerts a similar effect on media reporting (Mullainathan & Shleifer, 2005). As Hallin and Mancini (2004, p. 286) state, “commercial pressures can encourage media to differentiate themselves politically.” In the case of Austria, a formerly stable, institutionalized party-press parallelism has perhaps been replaced (or supplemented) by a more flexible, economically driven partisan bias determined by market interests.

There are at least four avenues for future research on partisan bias in media coverage of political messages. First, we need more comparative research on media gatekeeping, in particular in multiparty democracies, despite the challenges for such an endeavor in terms of data availability. Previous research has shown that news factors have different effects across countries (e.g., Helfer & Van Aelst, 2016). In addition, the relatively high ideological dispersion in the party and media systems and its substantial newspaper readership make Austria a particularly likely case for detecting a media partisanship in newspapers. Therefore, we cannot simply extrapolate our findings to other contexts. Following Hallin and Mancini’s (2004) categorization of media systems, we might expect that our findings are most likely to travel to other “democratic corporatist” countries in Western and Northern Europe with similar party and media systems. In contrast, we would expect partisan bias in media gatekeeping to matter less in liberal media systems with local, monopolistic media markets as in the United States or Canada (e.g., Hallin & Mancini, 2004). Research in these contexts may provide “least likely” cases for observing partisan gatekeeping.

Future research may also devote more attention to disentangling the sources of partisan gatekeeping bias. Puglisi and Snyder (2011) distinguish supply-side factors such as the political preferences of editors and journalists from demand side-driven explanations such as the preferences of their readership. Measures of supply-side-driven and demand-side-driven media partisanship are correlated, and our measurement approach focuses on the latter aspect. Yet, we cannot rule out that supply-side factors drive our results, and future studies should attempt to distinguish between both sources of partisan bias.

Such future research should also extend the time period under consideration. In this article, we examined the potentially heated campaign phase, yet the impact of partisan orientations may vary over the electoral cycle. Partisan bias may be higher during campaigns, as the media may try harder to appeal to readers on partisan grounds and journalists may be more biased themselves. Similarly, party communication may be particularly intense and conflictual in election periods compared to non-election times. On the other hand, the media may strive harder to maintain journalistic balance in the run-up to an election (e.g., Green-Pedersen et al., 2017; Van Aelst & De Swert, 2009; Walgrave & Van Aelst, 2006).

Another potential avenue for future research is to study different communication channels. Recent research indicates that partisan bias could even be stronger in private TV stations and digital media (Baum & Groeling, 2008; Groeling, 2008). These usually have higher levels of commercialization and may have to aim for niche markets, which could imply an overt marketing of ideological viewpoints and thus stronger effects of a partisan bias in message selection (e.g., Baum & Groeling, 2008). Similarly, future research should focus on different channels that parties use to get media attention for their messages. While we focus on press releases, social media platforms have become increasingly important for news production, news consumption, and the dissemination of news (Reuters Institute, 2016; Weeks & Holbert, 2013). Party messages distributed via social networks such as Twitter and Facebook may be more likely to be used by online media than traditional press releases; the content of such messages may also be less complex. Moreover, social media platforms are also attractive to political actors as they allow them to bypass media selection and to communicate directly with the electorate, thus increasing the speed and immediacy of party communication (e.g., Graham, Broersma, Hazelhoff, & Van’t Haar, 2013; Skovsgaard & Van Dalen, 2013). Given the broader potential audience of social network messages, their content may also differ from press releases. It would be interesting to study whether our findings also hold in these very different communication environments.

Finally, the impact of individual news factors should be examined more carefully. In our analysis, we pool different news factors in an index to analyze how a message’s news value conditions the impact of media partisanship. Yet, we do not address the question of which particular news factor is most likely to trigger this conditional effect; our robustness check (see supplemental Appendix D) seems to indicate that political roles may be particularly key. Recent research has also identified important differences in the explanatory power of news factors. For example, Helfer and Van Aelst (2016) show that surprise increases the chances that journalists report on a party press release, while conflict has no such effect. More nuanced tests of the contribution of individual news factors deserve greater attention in future research.

## Supplementary Material

Supplemental_materials.zip

## Notes

^1.^ In our empirical analysis, for example, journalists and editors could choose among about 45 press releases each day with peaks of up to 80 daily press releases.

^2.^ This argument also implies that the effect of news values is conditional on partisan bias: Media outlets focus on messages with higher news value, but they are most likely to focus on newsworthy messages from political actors they favor. For convenience, we only report the marginal effects in supplemental Appendix F.

^3.^ We discard press releases of the new liberal party (NEOS), which only gained seats after the election, and of other smaller parties without representation in parliament.

^4.^ By including them separately we account for differences in their news value according to their political role (see following discussion). The data set contains 83 press releases sent by two different politicians.

^5.^ The issue areas are economy, employment, social welfare and poverty, health care, pensions, family affairs, budget and taxes, agriculture, education, environment, law and order, individual rights and societal values, European integration, foreign affairs and defense, infrastructure, immigration, fighting political misconduct and corruption, government reforms and direct democracy.

^6.^ There are only a few newspapers with a Sunday edition. Thus, we also consider media reports published on Monday for those press releases published over the weekend.

^7.^ More information on the settings and the software is provided in supplemental Appendix A.

^8.^ Two party leaders were cabinet members; for them the indicator variable takes the value “2.”

^9.^ The exact question wording is, “There are several parties in Austria, each of which would like to receive your vote. Using the scale of 0–10, how likely is it that you would ever vote for each of the following parties?”

^10.^ The conditional hypothesis also implies a reverse argument: The effect of news factors on the success of press releases depends on partisan bias. The marginal effects plot in supplemental Appendix F provides evidence that partisan bias moderates the effect of a message’s news value.
