# Supplementary material for: Partisan Bias in Message Selection: Media Gatekeeping of Party Press Releases
Source: Polit Commun. 2017 Jul 3;34(3):367–84. doi: 10.1080/10584609.2016.1265619 (PMC5679709; doi:10.1080/10584609.2016.1265619)
Supplement: Supplemental_materials.zip [file UPCP_A_1265619_SM1603.zip › UPCP_A_1265619_SM1603/partisan_bias_in_message_selection_appendix.docx]

**Appendix A: Cheating detection software**

We use the software WCopyfind (version 4.1.4) developed by Lou Bloomfield (2014). This software allows users to compare documents (in our case: press releases and media reports) and detect similarities between texts. Results are presented in a tabular format and similarities between both texts are shown in a side-by-side format with similarities highlighted in color.

We use the following settings to run the software to identify successful press releases. First, we ignore punctuation, numbers, and capitalization, and set the language to Austrian-German. For each matching phrase, we also allow for one imperfection, meaning that one word in the matching phrase can differ (e.g. ‘in the election’ vs. ‘in the next election’). This accounts for minor editing changes by journalists. Because we aim to detect all potentially relevant press release-media report pairs, we set the shortest phrases that can match to ‘3’. If a phrase such as ‘in the election’ appears in both the press release and the media report, the pair of texts is thus saved for further investigation. This results in about 20,000 text pairs detected by the software.

For the manual coding process, we split this sample of 20,000 text pairs in two groups. The first group contains those pairs where a manual analysis of media reports, the AUTNES manual content analysis of media coverage in the 2013 general election (Eberl et al., 2015), has identified the sender of the press release as an active speaker in the headline, subtitle, or first paragraph of a media report. This group includes about 500 text pairs. These are observations that are likely to be successful because we know from the manual content analysis that the politician sending the press release was present in the media report. This sample is coded by two coders to distinguish between successful and unsuccessful press releases (see the definition in the manuscript).

The second, much larger group (N=19,863) contains the remaining text pairs detected by the software. These are not necessarily unsuccessful as the manual coding does not check for the full text of the media report and because individual politicians (senders of the press release) might be paraphrased using party labels. Therefore, we also check these pairs of text although the ex ante probability that this sample contains successful press releases is somewhat lower. We sort these text pairs by similarity, using the total number of perfect matches (identified by WCopyfind) as a yardstick. The total number of perfect matches indicates the sum of perfect matches in a press release-media report pair. For example, a score of ‘6’ indicates that both documents share a phrase of six words that is perfectly identical (or two phrases of three words that are identical). We decided to start the manual coding with those chunks of pairs where the similarity is highest and stop the coding process when the share of successful press releases falls below a certain threshold.

**Figure A.1: Successful press releases in manual coding by similarity in cheating detection software**


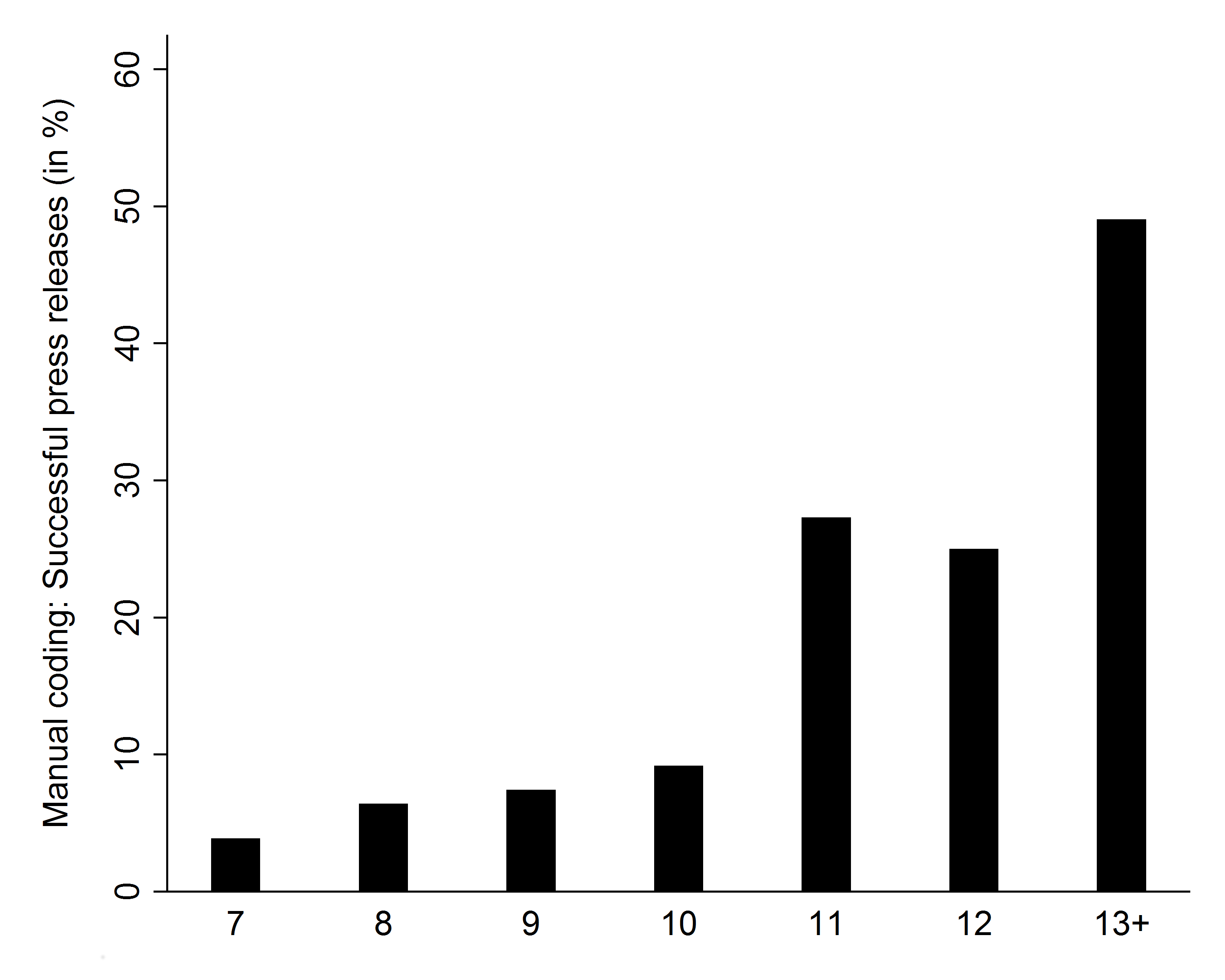


Note: Bars denote the average share of successful press releases identified in the manual coding process. The numbers below each bar denote the similarity score of each group as identified in the cheating detection software. For example, the press release-media report dyads in-group ‘7’ share a phrase with seven words (or two matched phrases, one with three and one with four words). Note that the group with ‘13+’ perfect matches contains dyads with 13 or more perfect matches.

Ultimately, we decided on a threshold of seven hits. To settle on this number, we proceeded as follow. First, we examined the results of this coding process as shown in Figure A.1. If the cheating detection software detected (sum of) strings of ten or more words, human coders classified about 10 per cent of these press releases as successful. The lower the similarity between the texts (as identified in the cheating detection software), the lower the share of successful press releases identified by human coders. We stopped the manual coding process after dyads with seven perfect matches as at that point the share of successful press releases is 3.8 per cent (i.e. 19 of 492 dyads were coded as successful). Assuming that the share of successful press releases in the manual coding is even lower as the similarity decreases even further, we deemed it unreasonable and unnecessary to continue the manual coding process.

**References:**

Bloomfield, Louis. (2014). *WCopyFind*. http://plagiarism.bloomfieldmedia.com/z-wordpress/software/wcopyfind/ (Release 4.1.4).

Eberl, Jakob-Moritz, Ramona Vonbun, Martin Haselmayer, Carina Jacobi, Katharina Kleinen-von Königslöw, Klaus Schönbach and Hajo Boomgaarden (2015). *AUTNES Manual Content Analysis of the 2013 Austrian National Election Coverage*. Version 1.4. Vienna: University of Vienna.

**Appendix B: Examples (extracts) of successful press releases**

**Table B.1: Examples (extracts) of successful press releases (English translation)**

| **Press release** | **Media Report** |
| --- | --- |
| **Fekter: SPÖ endangers middle class and prosperity**  […]  ‘The SPÖ endangers the middle class and prosperity.’ […] Regarding the Social Democrats’ plans for wealth taxes, Fekter notes: ‘Michael Spindelegger and the ÖVP want prosperity for all. In contrast, the SPÖ only aims to punish the people’s diligence and performance.’  […]  (7.9.2013)  <http://www.ots.at/presseaussendung/OTS_20130907_OTS0045/fekter-spoe-gefaehrdet-akut-mittelstand-und-wohlstand> | **ÖVP rails against SPÖ tax proposals**  ‘The SPÖ only aims to punish the people’s diligence and performance’, said Finance minister Maria Fekter (ÖVP) on Saturday in a comment on the SPÖ’s tax proposals. Several ÖVP politicians rejected those *Faymann taxes*, the overall theme being: prosperity and the middle class are endangered by property taxes.  […]  (*Kurier*, 8.9.2013) |
| **FPÖ-Kickl: Discussion on death penalty is ludicrous**  […]  ‘The discussion started by Frank Stronach to bring death penalty back into use is ludicrous and off target‘, Herbert Kickl stressed, reacting to statements by the party leader of Team Stronach. ‘If the death penalty is one of Team Stronach’s values, then good night’, Kickl said.  (5.9.2013)  <http://www.ots.at/presseaussendung/OTS_20130905_OTS0161/fpoe-kickl-todesstrafen-diskussion-ist-nur-skurril> | **Death penalty: Revolt against Stronach’s “Yes”**  […]  All other parties clearly rejected [Stronach’s] idea. For Minister of Justice Beatrix Karl (ÖVP) such a discussion was superfluous. […] The SPÖ spokesman for Justice, Hannes Jarolim, sees Stronach’s proposal in opposition to values in the European society. And for the FPÖ the discussion is ludicrous. ‘If the death penalty is one of Team Stronach’s values, then good night’, party chairman Herbert Kickl said.  […]  (*Die Presse*, 6.9.2013) |

**Table B.2: Examples (extracts) of successful press releases (German original)**

| **Press release** | **Media Report** |
| --- | --- |
| **Fekter: SPÖ gefährdet akut Mittelstand und Wohlstand**  […]  „Die SPÖ gefährdet akut den Mittelstand und den Wohlstand. […] Zu den Besteuerungsplänen der Sozialisten unterstreicht Fekter: „Michael Spindelegger und die ÖVP wollen Wohlstand für alle. Der SPÖ geht es nur darum, Leistung und Fleiß zu bestrafen.“  […]  (7.9.2013)  <http://www.ots.at/presseaussendung/OTS_20130907_OTS0045/fekter-spoe-gefaehrdet-akut-mittelstand-und-wohlstand> | **ÖVP wettert erneut gegen SP-Steuerpläne**  „Der SPÖ geht es nur darum, Leistung und Fleiß zu bestrafen“, sagte ÖVP-Finanzminister Maria Fekter am Samstag zu den Steuerplänen der SPÖ. Mehrere VP-Mandatare meldeten sich gegen die Faymann-Steuern zu Wort, der rote Faden: Wohl- und Mittelstand seien durch Vermögenssteuern gefährdet.  […]  (*Kurier*, 8.9.2013) |
| **FPÖ-Kickl: Todesstrafen-Diskussion ist nur skurril**  […]  „Die von Frank Stronach angefangenen Diskussion um die Wiedereinführung der Todesstrafe ist skurril und geht am Thema vorbei“, betonte der freiheitliche Generalsekretär NAbg. Herbert Kickl in einer Reaktion auf diesbezügliche Aussagen des Team-Stronach Chefs. „Wenn die Todesstrafe einer der Werte des Team Stronach ist, dann Gute Nacht“, so Kickl.  […]  (5.9.2013)  http://www.ots.at/presseaussendung/OTS_20130905_OTS0161/fpoe-kickl-todesstrafen-diskussion-ist-nur-skurril | **Todesstrafe: Revolte gegen Stronachs Ja**  […]  Entsprechend eindeutig fiel auch die Ablehnung der anderen Parteien aus. Justizministerin Beatrix Karl (ÖVP) erklärte, darüber erübrige sich jede Diskussion. […] Für SPÖ-Justizsprecher Hannes Jarolim steht Stronach konträr zu den Werten der europäischen Gesellschaft. Und für die FPÖ ist die Diskussion skurril. „Wenn die Todesstrafe einer der Werte des Teams Stronach ist, dann gute Nacht“, so Generalsekretär Herbert Kickl.  […]  (*Die Presse*, 6.9.2013) |

**Appendix C: Multilevel vs. clustered standard errors (logistic regression)**

|  | Model 1 (ML) | Model 1 (CL) | Model 2 (ML) | Model 2 (CL) |
| --- | --- | --- | --- | --- |
| News value index | 0.364*** | 0.325*** | -0.355 | -0.364+ |
|  | (0.077) | (0.068) | (0.283) | (0.211) |
| Readership party orientation | 0.571*** | 0.515** | 0.0856 | 0.0435 |
|  | (0.173) | (0.157) | (0.251) | (0.222) |
| Readership party orientation X News value index |  |  | 0.197** | 0.188** |
|  |  |  | (0.075) | (0.060) |
| Female | -0.386+ | -0.240 | -0.356 | -0.214 |
|  | (0.225) | (0.193) | (0.223) | (0.194) |
| Time PR sent | -0.00140+ | -0.00116+ | -0.00144* | -0.00123* |
|  | (0.001) | (0.001) | (0.001) | (0.001) |
| Date | -0.0235** | -0.0181*** | -0.0235*** | -0.0184*** |
|  | (0.008) | (0.005) | (0.006) | (0.005) |
| PR based on external event | -0.104 | 0.0617 | -0.0932 | 0.0681 |
|  | (0.203) | (0.185) | (0.201) | (0.185) |
| PR with press conference | 1.537*** | 1.204*** | 1.479*** | 1.169*** |
|  | (0.295) | (0.207) | (0.292) | (0.210) |
| Number of articles per newspaper | 0.000948*** | 0.000811*** | 0.000948*** | 0.000818*** |
|  | (0.0001) | (0.0001) | (0.0001) | (0.0001) |
| Text length | 0.00326*** | 0.00252*** | 0.00327*** | 0.00253*** |
|  | (0.001) | (0.001) | (0.001) | (0.001) |
| Constant | 452.1** | 347.6** | 453.6*** | 355.5*** |
|  | (154.501) | (106.661) | (120.557) | (105.107) |
| Based on 1,496 cluster / press releases |  |  |  |  |
| Sigma (SD) | 0.528***  (0.076) |  | 0.511**  (0.076)* |  |
| Observations | 11968 | 11968 | 11968 | 11968 |
| Log likelihood | -1451.1 | -1553.4 | -1447.6 | -1545.9 |

Standard errors in parentheses

Party fixed effects included, but not reported in the table.

^+^ *p* < 0.1, ^*^ *p* < 0.05, ^**^ *p* < 0.01, ^***^ *p* < 0.001

**Appendix D: Excluding one news factor at a time (logistic regression)**

|  | | | News value index | | | |  |
| --- | --- | --- | --- | --- | --- | --- | --- |
|  | w/o  surprise | w/o powerful elites | | w/o relevance | w/o  conflict | w/o negativity | |
| **News value index** | **0.399^***^** | **0.121^+^** | | **0.423^***^** | **0.314^***^** | **0.404^***^** | |
|  | **(0.074)** | **(0.071)** | | **(0.079)** | **(0.082)** | **(0.076)** | |
| Readership party orientation | 0.515^**^ | 0.515^**^ | | 0.515^**^ | 0.515^**^ | 0.515^**^ | |
|  | (0.158) | (0.157) | | (0.158) | (0.157) | (0.157) | |
| Female | -0.227 | -0.323^+^ | | -0.214 | -0.249 | -0.249 | |
|  | (0.191) | (0.194) | | (0.192) | (0.195) | (0.192) | |
| Time PR sent | -0.00118^*^ | -0.00104 | | -0.00114^+^ | -0.00115^+^ | -0.00125^*^ | |
|  | (0.001) | (0.001) | | (0.001) | (0.001) | (0.001) | |
| Date | -0.0173^**^ | -0.0187^***^ | | -0.0178^**^ | -0.0185^***^ | -0.0189^***^ | |
|  | (0.005) | (0.005) | | (0.005) | (0.006) | (0.005) | |
| PR based on external event | 0.0803 | -0.0246 | | 0.0614 | 0.0668 | 0.00798 | |
|  | (0.183) | (0.185) | | (0.184) | (0.187) | (0.184) | |
| PR with press conference | 1.205^***^ | 1.211^***^ | | 1.164^***^ | 1.259^***^ | 1.157^***^ | |
|  | (0.206) | (0.211) | | (0.204) | (0.211) | (0.210) | |
| Number of articles per newspaper | 0.000812^***^ | 0.000806^***^ | | 0.000813^***^ | 0.000809^***^ | 0.000812^***^ | |
|  | (0.0001) | (0.0001) | | (0.0001) | (0.0001) | (0.0001) | |
| Text length | 0.00250^***^ | 0.00244^***^ | | 0.00250^***^ | 0.00229^***^ | 0.00236^***^ | |
|  | (0.001) | (0.001) | | (0.001) | (0.001) | (0.001) | |
| Constant | 332.9^**^ | 360.8^***^ | | 342.0^**^ | 355.4^**^ | 364.5^***^ | |
|  | (106.826) | (107.223) | | (107.385) | (108.303) | (106.117) | |
| Observations | 11968 | 11968 | | 11968 | 11968 | 11968 | |
| Log likelihood | -1548.1 | -1574.0 | | -1546.0 | -1561.0 | -1548.3 | |

Clustered standard errors (for press releases) in parentheses (1,496 clusters)

Party fixed effects included but not reported in the table.

^+^ *p* < 0.1, ^*^ *p* < 0.05, ^**^ *p* < 0.01, ^***^ *p* < 0.001

**Appendix E: Effect of partisan bias without SPÖ, ÖVP and BZÖ**

|  | Model 1 (reported in the manuscript) | Model 2 (excluding SPÖ, ÖVP & BZÖ) |
| --- | --- | --- |
| News value index | 0.325^***^ | 0.129 |
|  | (0.068) | (0.108) |
| **Readership party orientation** | **0.515^**^** | **0.439^*^** |
|  | **(0.157)** | **(0.190)** |
| Female | -0.240 | -0.0315 |
|  | (0.193) | (0.350) |
| Time PR sent | -0.00116^+^ | -0.00209^+^ |
|  | (0.001) | (0.001) |
| Date | -0.0181^***^ | -0.0130 |
|  | (0.005) | (0.008) |
| PR based on external event | 0.0617 | -0.159 |
|  | (0.185) | (0.221) |
| PR with press conference | 1.204^***^ | (0.284) |
|  | (0.207) | 0.691 |
| Number of articles per newspaper | 0.000811^***^ | 0.00115^***^ |
|  | (0.0001) | (0.0002) |
| Text length | 0.00252^***^ | 0.00120 |
|  | (0.001) | (0.001) |
| Constant | 347.6^**^ | 249.3 |
|  | (106.661) | (164.009) |
| Observations | 11968 | 5296 |
| Log likelihood | -1553.4 | -578.5 |

Clustered standard errors (for press releases) in parentheses (Model 1: 1,496 clusters; Model 2: 662 clusters)

Party fixed effects included but not reported in the table.

^+^ *p* < 0.1, ^*^ *p* < 0.05, ^**^ *p* < 0.01, ^***^ *p* < 0.001

**Appendix F: Marginal effect of news value conditional on partisan bias**

**
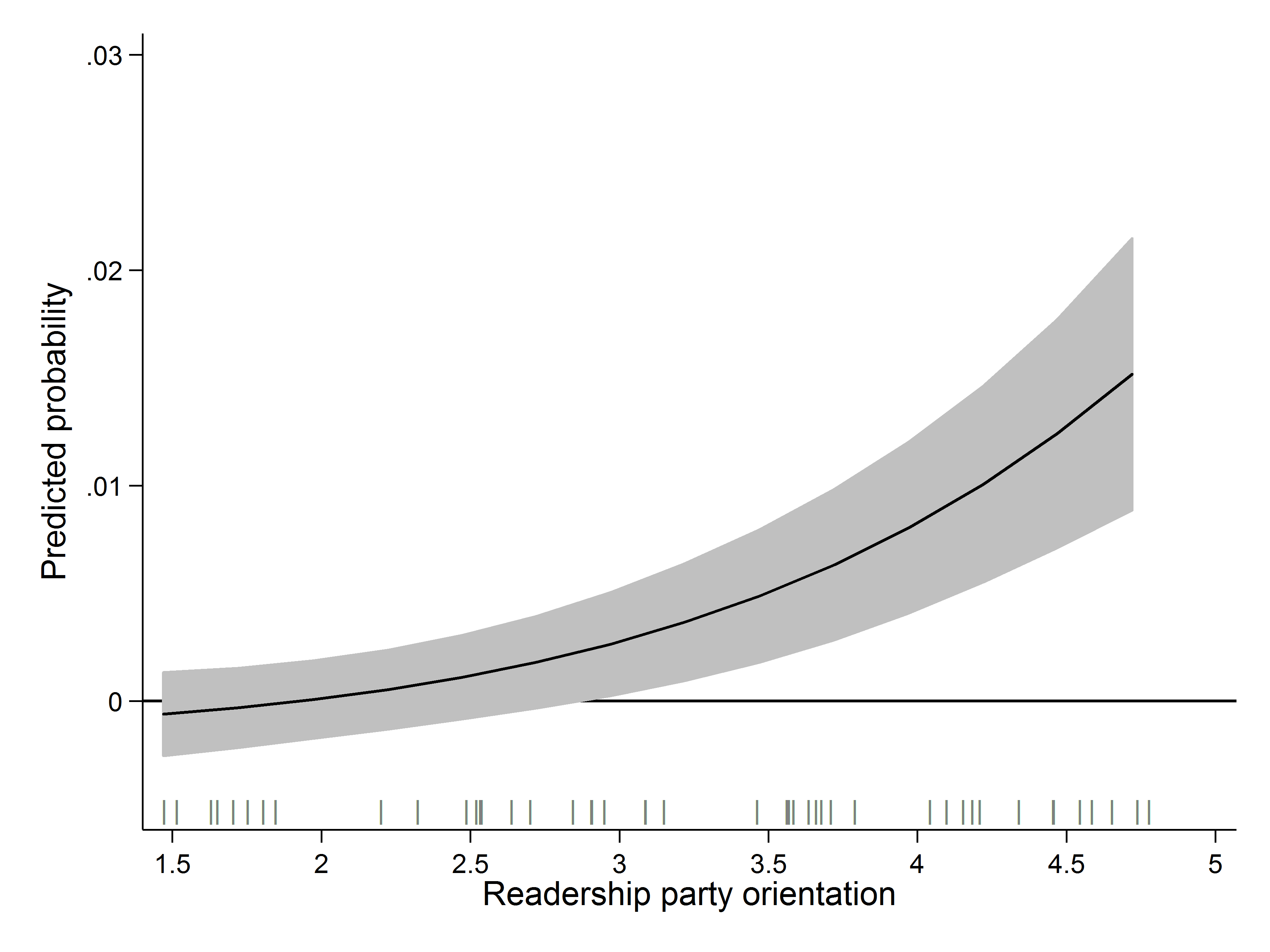
**

Notes: Marginal effects are based on Model 2 (Table 1) holding all other variables at their mean or mode. Shaded areas indicate 95% confidence intervals. The vertical bars (pipes) report the distribution of the moderating variable.
